# *SYNE1* Mutation Is Associated with Increased Tumor Mutation Burden and Immune Cell Infiltration in Ovarian Cancer

**DOI:** 10.3390/ijms241814212

**Published:** 2023-09-18

**Authors:** Laura M. Harbin, Nan Lin, Frederick R. Ueland, Jill M. Kolesar

**Affiliations:** 1 Division of Gynecologic Oncology, Department of Obstetrics and Gynecology, University of Kentucky Markey Cancer Center, 800 Rose Street, Lexington, KY 20536-0596, USA; 2 Department of Pharmacy Practice and Science, University of Kentucky College of Pharmacy, 760 Press Avenue, Lexington, KY 40536-0596, USA

**Keywords:** *SYNE1*, ovarian cancer, immunotherapy, tumor mutation burden

## Abstract

*SYNE1*, a nuclear envelope protein critical for cellular structure and signaling, is downregulated in numerous malignancies. *SYNE1* alterations are found in 10% of gynecologic malignancies and 5% of epithelial ovarian cancers. Previous studies demonstrated an association between *SYNE1* mutation, increased tumor mutation burden (TMB), and immunotherapy response. This study evaluates the *SYNE1* mutation frequency, association with TMB, and downstream effects of *SYNE1* mutation in ovarian cancer. Genetic information, including whole-exome sequencing, RNA analysis, and somatic tumor testing, was obtained for consenting ovarian cancer patients at an academic medical center. Mutation frequencies were compared between the institutional cohort and The Cancer Genome Atlas (TCGA). Bioinformatics analyses were performed. In our cohort of 50 patients, 16 had a *SYNE1* mutation, and 15 had recurrent disease. Median TMB for *SYNE1* mutated patients was 25 compared to 7 for *SYNE1* wild-type patients (*p* < 0.0001). Compared to the TCGA cohort, our cohort had higher *SYNE1* mutation rates (32% vs. 6%, *p* < 0.001). Gene expression related to immune cell trafficking, inflammatory response, and immune response (z > 2.0) was significantly increased in *SYNE1* mutated patients. *SYNE1* mutation is associated with increased TMB and immune cell infiltration in ovarian cancer and may serve as an additional biomarker for immunotherapy response.

## 1. Introduction

Ovarian cancer (OC) is the fifth most common cause of cancer-related deaths in the United States and is the deadliest of all gynecologic malignancies [1,2]. A woman’s risk of developing ovarian cancer during her lifetime is approximately 1 in 78, and her risk of dying is about 1 in 100 [3]. Unfortunately, we still lack effective screening for ovarian cancer, leading most women to receive their diagnosis at an advanced stage [4].

Recent developments in precision medicine and somatic tumor sequencing have offered a greater understanding of the genomic signatures of various tumors. Genomic instability is a hallmark of ovarian cancer and results in higher tumor mutation counts compared to other gynecologic malignancies [5,6]. One study by Chava and Gupta found that most ovarian cancers harbored mutational burdens of 30–40 alterations, including missense mutations, in-frame mutations, copy number alterations, and loss of heterozygosity (LOH) [5]. The most commonly mutated genes in ovarian cancer include *TP53*, *PIK3CA*, *KRAS*, *KMT2C*, *PTEN*, and *ARID1A* [5,7]. *SYNE1*, a less commonly mutated gene, had notable alterations in 10% of gynecologic malignancies and 5% of epithelial ovarian cancers [5,8].

The *SYNE1* gene encodes a nuclear envelope protein, nesprin-1, critical for connecting the nucleus to the cytoskeleton [5,9,10,11]. Nesprin-1 deficiency leads to abnormal nuclear morphology, cell motility, and cytoskeleton organization [9,10,12,13]. Genomic reports identify the downregulation of *SYNE1* in ovarian cancer and other malignancies, but the consequence of this is not well understood [5,14,15,16,17,18]. Sur et al. identified nesprin-1 as an important protein in the DNA repair complex, specifically interacting with MSH2 and MSH6 in the MutSa complex required for mismatch repair (MMR) [9]. They also found that cells deficient in nesprin-1 were associated with concurrent deficiency of wild-type MSH2 and MSH6, suggesting a potential association with MMR [9]. A study in renal cell carcinoma identified *SYNE1* as a marker for high tumor mutation burden (TMB) and response to immune checkpoint inhibitors (ICI) [19].

This study evaluates the *SYNE1* mutation frequency, its association with tumor mutation burden, and the downstream effects of *SYNE1* mutations in ovarian cancer.

## 2. Results

### 2.1. Demographics

Our population included 50 patients diagnosed with ovarian cancer; the demographics are shown in Table 1. The median age was 64 years, and the median BMI was 27 kg/m^2^. High-grade serous histology was most common and present in 66% of patients. Other histologic subtypes included the following: endometrioid carcinoma (8%), granulosa cell tumor (6%), carcinosarcoma (4%), mucinous carcinoma (2%), and other malignancies (14%). Most patients were diagnosed at advanced stage with 45% of patients diagnosed with stage III disease, and 19% of patients diagnosed with stage IV disease. Approximately 60% of patients were from Appalachian counties, which reflects the proportion of patients from this region at our institution.

Of the 50 patients, 16 had a *SYNE1* mutation. There was no difference between the *SYNE1* mutated and *SYNE1* wild-type (WT) groups in terms of age, BMI, race, tumor histology, or stage at diagnosis (Table 1). There was also no difference between groups regarding Appalachian status or metropolitan status. Overall, 15 of the 50 patients experienced disease recurrence. In the *SYNE1* WT group, 12 of the 34 patients (35%) recurred compared to 3 of the 12 (19%) *SYNE1* mutant patients. However, this difference was not significant (*p* = 0.3).

### 2.2. Whole Exome Sequencing

**Figure 1:**
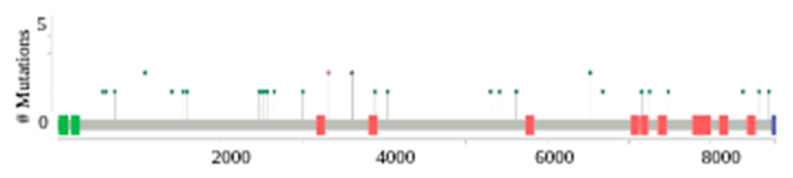
*SYNE1* lollipop plot for mutations in 50 ovarian cancer patients at The Markey Cancer Center. Missense mutations are represented in green, truncating mutations in black, and all other mutation types in red (excluding fusions and in-frame deletions or insertions). (aa: amino acid).

*SYNE1* mutations were detected via whole exome sequencing (WES) and are depicted in the lollipop plot (Figure 1). Thirty-three unique mutations were identified, including 28 missense mutations, 3 non-sense mutations, and 2 intron mutations. All the detected mutations were caused by a single nucleotide substitution, as shown in Table S1. Moreover, 9 of the 16 patients had more than one single nucleotide substitution detected via WES.

**Figure 2:**
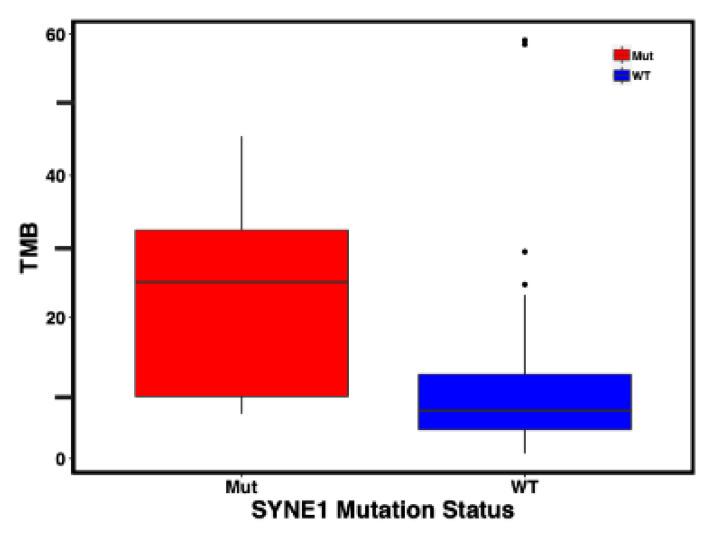
Comparison of tumor mutation burden (TMB) between *SYNE1* mutated and *SYNE1* wild-type patients. When treated as a continuous variable *SYNE1* mutated patients had a median TMB of 25 compared to median TMB of 7 for *SYNE1* WT patients (*p* < 0.0001). (TMB: tumor mutation burden (mutations per Megabase), Mut: mutated, WT: wild-type).

Given the previous association of *SYNE1* mutation with tumor mutation burden (TMB), we compared the TMB status of *SYNE1* wild-type (WT) to *SYNE1* mutated patients. When TMB was treated as a continuous variable, *SYNE1* mutated patients had a median TMB of 25 mutations per megabase of DNA (standard deviation (SD) 13.5) compared to median TMB of 7 (SD 14.7) for *SYNE1* WT patients (*p*-value < 0.0001) (Figure 2). When comparing TMB as a categorical variable, we defined TMB greater than or equal to 10 mutations per megabase (≥10 mut/Mb) as TMB-high. *SYNE1* mutated patients were more likely to be categorized as TMB high (*p*-value = 0.02) (Table 2). Whether treated as a continuous or categorical variable, *SYNE1* mutation was associated with a TMB-high status (Figure 2, Table 2).

Previous studies have suggested that *SYNE1* plays a role in mismatch repair (MMR) via interactions with MSH2 and MSH6. We compared microsatellite instability status (MSI) between *SYNE1* mutated and *SYNE1* wild-type patients as a surrogate for MMR deficiency. For the comparison of MSI as a categorical variable, MSI greater than 20 was considered MSI-high. No patients in the *SYNE1* mutated or *SYNE1* WT groups were classified as MSI-high (*p*-value not applicable) (Table 2).

In our population of 50 ovarian cancer patients treated at the Markey Cancer Center, 16 patients (32%) exhibited the *SYNE1* mutant phenotype. To determine how our incidence of *SYNE1* mutation compared to the general population, we compared our cohort to TCGA PanCancer Atlas ovarian serous cystadenocarcinoma cohort. The incidence of *SYNE1* mutation in the cohort of TCGA was 5% compared to 32% in the MCC cohort (*p*-value < 0.001) (Table 3). Of the 18 genes compared between the two cohorts, all but 4 genes were mutated at a significantly higher rate in the MCC cohort (Table 3). Notable genes with a statistically higher incidence in the MCC cohort included *BRCA1*, *BRCA2*, *PIK3CA,* and *ARID1A*. This statistical significance remained after correcting for multiple comparisons.

To determine if *SYNE1* mutations co-occur with other genetic mutations, a Fisher’s exact test and co-occurrence analysis were performed (Table 4). We found that *USH2A* and *KMT2D* mutations significantly co-occurred with *SYNE1* mutations with *p*-values of 0.04 and 0.007, respectively. After correction for multiple comparisons, these values lost significance with q-values of 0.3 and 0.1, respectively. Other genes associated with mismatch repair and homologous recombination repair, including *MSH2, MSH6, BRCA1, and BRCA2*, were not found to be significantly co-mutated with *SYNE1*.

### 2.3. RNA Analysis

**Figure 3:**
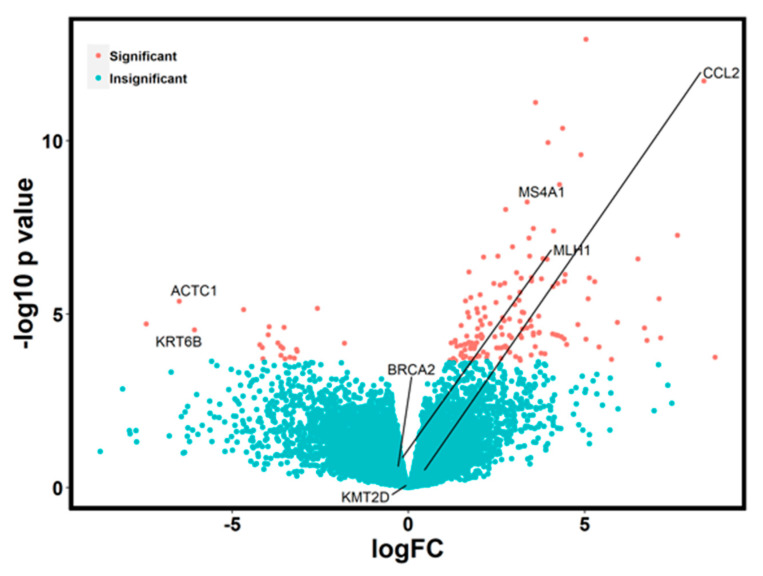
Differential gene expression between *SYNE1* mutated and *SYNE1* wild-type patients.

Given the role of *SYNE1* in gene stability and gene expression, we used RNA sequencing analysis via Qiagen’s Ingenuity Pathway Analysis (IPA) to compare gene expression between *SYNE1* mutated and wild-type patients. Our goal was to better understand the role *SYNE1* plays in malignant cells. Analysis revealed significantly different gene expressions in 168 genes between the *SYNE1* mutated and *SYNE1* WT patients (Figure 3). The top 10 upregulated and downregulated genes in *SYNE1* mutated patients are listed in Table 5. Several of the upregulated genes play prominent roles in immune activation, cell signaling, transcriptional regulation, and apoptosis regulation. In contrast, several of the downregulated genes are important in cell motility, structural integrity, and cytoskeleton signaling.

**Figure 4:**
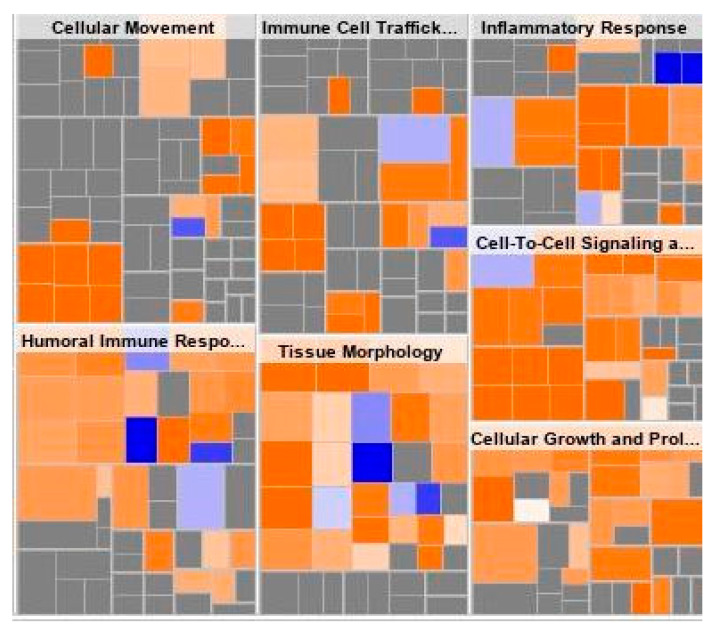
Differential gene expression and pathway analysis between *SYNE1* mutant and *SYNE1* wild-type patients. The size of the box denotes the -log (*p*-value). The color of the boxes correlates with the z-score with the intensity of blue representing z < 0 and orange z > 0. Significantly different gene expression is noted for leukocyte migration, recruitment of leukocytes, myeloid cells, and granulocytes, and localization of myeloid cells. There are also significant increases in immune cell trafficking, inflammatory response, and humoral immune response.

Heat maps were used to better depict the change in RNA expression among *SYNE1* mutated patients. As demonstrated in Figure 4, *SYNE1* mutated patients had notable differences in gene expression related to immune cell trafficking, inflammatory response, and humoral immune response.

In particular, *SYNE1* mutated patients demonstrated gene expression associated with increased leukocyte migration, increased recruitment of myeloid cells, and increased localization of myeloid cells. Additionally, heat maps indicate that *SYNE1* mutated patients exhibit differential expression of genes involved in cellular movement, cell-to-cell signaling, and cellular growth and proliferation.

## 3. Discussion

Immune checkpoint inhibitors have revolutionized the treatment of most solid tumors. However, their activity in ovarian cancer is minimal. Single-agent PD-1 or PD-L1 inhibitors in unselected, advanced recurrent ovarian cancer have yielded poor response rates of approximately 10% [20,21]. TMB is a predictive biomarker for immune checkpoint inhibitor response, with FDA-approved pembrolizumab for patients with advanced solid tumors with a TMB of 10 or higher, although no ovarian cancer patients were included in this registrational study [22,23,24]. Consistent with what is observed in renal cell cancer, *SYNE1* mutation is associated with an increased TMB in ovarian cancer as well, with 66% of our patients with a *SYNE1* mutation having a TMB ≥ 10 [19]. This appears higher than previously reported rates of TMB-high ovarian cancer ranging from 4–15% with incidence varying by histologic subtype [25]. A prior report demonstrating the association of nesprin-1 loss and increased double-strand DNA breaks suggests that *SYNE1* mutation could lead to increased TMB [9].

MSI-high and MMR deficiency were not associated with *SYNE1* mutation in this study. MSI-high status is commonly driven by mutations in mismatch repair genes, including *MSH2* and *MSH6*. Nesprin-1 is thought to localize wild-type *MSH2* and *MSH6* to the nucleus for participation in DNA repair. Given this, the co-mutation of genes in the same regulatory pathway could result in synthetic lethality rather than tumorigenesis [9]. This further supports the importance of nesprin-1 in DNA repair. Importantly, *SYNE1* mutation was associated with increased immune cell infiltration, which suggests an improved response to immunotherapy.

We also demonstrate that *MS4A1* gene expression is significantly upregulated in *SYNE1*-mutated patients. This gene codes for the B-lymphocyte surface molecule CD20 play a role in the development and differentiation of B-cells into plasma cells. *MS4A1* expression is positively correlated with CD4+ and CD8+T cell infiltration in ovarian cancer [26,27]. Additionally, higher CD20+ B cell and CD8+T cell counts are associated with improved response to checkpoint inhibitors in women with ovarian cancer [28]. Consistently, our pathway analysis demonstrates alterations in immune pathways, with immune cell trafficking, humoral immune response, and inflammatory response significantly upregulated in *SYNE1* mutant ovarian cancer. Since ovarian cancer is generally considered a “cold” tumor with a low TMB and lack of CD8+ T cells, this provides additional support for the evaluation of *SYNE1* as a biomarker of immunotherapy response [29].

We observed an increased frequency of *SYNE1* mutations in women with ovarian cancer residing in Kentucky when compared to women in other parts of North America. However, *SYNE1* mutation was not associated with specific clinical characteristics or outcomes. Like *SYNE1,* our population also demonstrated more frequent mutations in *BRCA1, BRCA2, PIK3CA*, and *PTEN. KRAS, ARID1A, MSH2*, and *MSH6* had similar mutation rates between the groups, and *TP53* was less frequently mutated. We anticipate this is related to differences between our population and the TCGA population and is likely multifactorial. TCGA population almost entirely comprises high-grade serous and serous cystadenocacinoma, while our population contained multiple additional histologic sub-types. Additionally, several studies have demonstrated increased mutation frequency among the Appalachian population [30,31,32]. This is observed across cancer types in this region and suggests a potential association with environmental, socioeconomic, and genetic factors [30,31,32].

Strengths of this investigation include a population with multiple histologic sub-types, which, to our knowledge, are not represented in currently available broad genomic databases. We also linked patient data with whole exome sequencing and RNA sequencing to perform a comprehensive assessment of the clinical and genomic landscape of *SYNE1* mutated patients. The limitations of this study include our small sample size of Kentucky ovarian cancer patients which limits generalizability and may be too small to detect clinical characteristics associated with *SYNE 1* mutations. In addition, we are unable to determine the etiology of the increased mutation rate. We also compared our cohort to the TCGA potentially introducing inconsistencies in sequencing and bioinformatics processing, although we employed conservative variant calling which would bias towards under-calling variants [32].

In conclusion, women with ovarian cancer and residing in Kentucky are more likely to have a *SYNE1* mutation than women residing in other parts of North America. *SYNE1* is associated with both an increased tumor mutation burden and overexpression of *MS4A1*. Taken together, there is evidence that *SYNE1* may be a predictive biomarker for immunotherapy response in ovarian cancer.

## 4. Materials and Methods

### 4.1. Study Population and Design

In the Commonwealth of Kentucky, all patients diagnosed or treated for cancer are confidentially reported to the Kentucky Cancer Registry (KCR) by state statute (KRS 214.556). Since its inception in 2012, the KCR has collected demographic, clinical, and genetic information from patients to improve cancer screening, prevention, diagnosis, and treatment.

The Markey Cancer Center (MCC) at the University of Kentucky (UK), the only NCI-designated cancer center in the state, participates in the KCR in addition to the Oncology Research Information Exchange Network (ORIEN). The ORIEN network includes 19 cancer centers across the United States and allows for shared data and tissue repositories for cancer research. Patients treated at ORIEN alliance cancer centers can enroll in a prospective cohort study, Total Cancer Care (TCC)**^®^**. TCC utilizes a standardized protocol to collect each patient’s genetic information, including whole-exome sequencing, RNA analysis, germline testing, and somatic tumor testing.

Eligible patients treated at MCC were invited to participate in this TCC study. All patients over the age of 18 with a diagnosis of cancer were eligible. Between February 2018 and August 2019, 50 patients diagnosed with ovarian cancer with germline and somatic whole exome sequencing were enrolled and assigned a TCC identification number. The TCC ID was linked to the identical patient in the KCR to sync genomic data with clinical data. The data was then assimilated and deidentified via the Cancer Research Informatics Shared Research Facility at the Markey Cancer Center. This study was IRB approved via the University of Kentucky (IRB# 50767), and all patients provided informed consent before enrollment in this study.

Demographic variables were obtained via the linked KCR. Patients were classified based on primary tumor histology. The patient’s documented zip code and county of residence were used to separate patients into groups based on urban setting and Appalachian status. The Cancer Genome Atlas (TCGA) PanCancer Atlas dataset was used to compare gene mutation frequency between TCGA population and the MCC population. A total of 523 ovarian cancer patients in the Ovarian Serous Cystadenocarcinoma cohort (TCGA, PanCancer Atlas) were compared to the 50 patients in our study population, and the link is included here: https://www.cbioportal.org/study/summary?id=ov_tcga_pan_can_atlas_2018, accessed on 12 April 2023. Sequencing methods for whole exome sequencing and RNA sequencing have been previously described [32].

### 4.2. Bioinformatics

Raw whole exome sequencing reads were processed through the bioinformatics pipeline developed by M2Gen. This process performs alignment, discovery, quality control, and evaluation procedures. Adaptor sequences were first trimmed via Bbduk software (https://jgi.doe.gov/data-and-tools/software-tools/bbtools/, accessed on 16 August 2023) with paired-end read option. Reads were then mapped to the human genome using BWA-mem using GRCh38 human genome as reference and paired-end option. Duplication and quality of mapping were investigated and filtered using Picard Mark Dups and recalibration. Single sample NSV and indels were further called and annotated using GATK haplotyper and funcotator. This was followed by tumor mutation burden analysis. Last, the quality of single sample copy number variants and indels was evaluated using Picard and GATK quality control pipeline, including corresponding contamination concordance analysis.

### 4.3. Statistical Analysis

Descriptive analysis of clinical variables and cancer-related prognostic factors was conducted, including age, BMI, Appalachian status, tobacco use, tumor grade, tumor stage, and cancer therapy. Comparison of continuous and categorical variables was conducted using Student *t*-test, Chi-square test, or Fisher’s exact test, respectively. Tumor mutation burden was calculated with the Wilcoxon rank sum test. TMB was calculated by counting the non-synonymous somatic mutations per megabase in the coding region of our captured data. Single nucleotide variants, missense mutations, nonsense mutations, and read-through mutations were included in the calculation of TMB.

## Figures and Tables

**Table 1:** Demographics of Markey Cancer Center ovarian cancer patients. Comparison of *SYNE1* wild-type with *SYNE1* mutated patient demographics. (WT: wild-type, mut: mutated, BMI: body mass index, IQR: inter-quartile range, NOS: not otherwise specified).

Variable	Total Population N = 50	*SYNE1* WT N = 34 (% of Pop)	*SYNE1* Mut N = 16 (% of Pop)	*p*-Value
Age	64 years (IQR 53, 72)	63 (IQR 54, 70)	65 (IQR 52, 72)	0.9
BMI	27 kg/m^2^ (IQR 24, 32)	27 kg/m^2^ (IQR 23, 31)	28 kg/m^2^ (IQR 24, 38)	0.2
Race				
White	50 (100%)	34 (100%)	16 (100%)	1
Stage				
I	7 (14%)	4 (12%)	3 (19%)	>0.9
II	8 (16%)	5 (15%)	3 (19%)	
III	19 (38%)	12 (35%)	7 (44%)	
IV	8 (16%)	5 (15%)	3 (19%)	
Unknown	8 (16%)	8 (23%)	0 (0%)	
Histology				
Carcinosarcoma	2 (4%)	2 (5.9%)	0 (0%)	0.9
Endometrioid	4 (8%)	2 (5.9%)	2 (12%)	
Granulosa cell tumor	3 (6%)	3 (7.8%)	0 (0%)	
High grade serous	33 (66%)	21 (62%)	12 (75%)	
Mucinous	1 (2%)	1 (2.9%)	0 (0%)	
Other	7 (14%)	5 (15%)	2 (12%)	
Urban setting				
Metropolitan county	14 (28%)	11 (32%)	3 (19%)	0.5
Non-metropolitan	36 (72%)	23 (68%)	13 (81%)	
Appalachia status				
Appalachian county	32 (64%)	22 (65%)	10 (62%)	0.9
Non-Appalachian	18 (36%)	12 (35%)	6 (38%)	
Insurance provider				
Medicare	22 (44%)	13 (38%)	9 (56%)	0.14
Private insurance	21 (42%)	17 (50%)	4 (25%)	
Medicaid	4 (8%)	3 (8.8%)	1 (6.2%)	
Not insured, self-pay	1 (2%)	0 (0%)	1 (6.2%)	
Insurance, NOS	1 (2%)	1 (2.9%)	0 (0%)	
Unknown	1 (2%)	0 (0%)	1 (6.2%)	
Smoking status				
Non-smoker	30 (62%)	21 (66%)	9 (56%)	0.5
Smoker	18 (38%)	11 (34%)	7 (44%)	
Recurrence	15 (30%)	12 (35%)	3 (19%)	0.3

**Table 2:** Comparison of tumor mutation burden and microsatellite instability status as categorical variables in *SYNE1* wild-type and *SYNE1* mutated patients. (TMB: tumor mutation burden, MSI: microsatellite instability).

	*SYNE1* WT	*SYNE1* Mutant	*p*-Value
TMB < 10	22	5	0.02
TMB ≥ 10	10	10	
MSI < 20	32	15	N/a
MSI > 20	0	0	

**Table 3:** Comparison of mutation frequency between MCC and TCGA PanCancer Atlas ovarian cancer patients. (TCGA: The Cancer Genome Atlas, MCC: Markey Cancer Center).

	TCGA Frequency (%) N = 523	MCC Frequency (%) N = 50	q-Value
Higher mutation frequency in MCC			
*TTN*	110 (21%)	40 (80%)	<0.001 *
*MUC16*	41 (8%)	25 (50%)	<0.001 *
*CSMD3*	38 (7%)	11 (22%)	<0.001 *
*USH2A*	32 (6%)	14 (28%)	<0.001 *
*RYR2*	28 (5%)	13 (26%)	<0.001 *
*SYNE1*	26 (5%)	16 (32%)	<0.001 *
*BRCA1*	18 (3%)	11 (22%)	<0.001 *
*BRCA2*	15 (2.9%)	13 (26%)	<0.001 *
*KMT2D*	9 (1.9%)	18 (36%)	<0.001 *
*PIK3CA*	8 (1.5%)	10 (20%)	<0.001 *
*PTEN*	7 (1.3%)	10 (20%)	<0.001 *
*ARID1A*	4 (0.8%)	10 (20%)	0.032 *
*PIK3R1*	1 (0.2%)	4 (8%)	0.022 *
*MSH2*	4 (0.8%)	4 (8%)	0.090
*MSH6*	3 (0.6%)	1 (2%)	0.140
*KRAS*	6 (1.1%)	2 (4%)	0.149
Lower mutation frequency in MCC			
*TP53*	373 (71%)	25 (50%)	0.01 *
*PPP2R1A*	5 (1%)	0 (0%)	1

* denotes statistical significance.

**Table 4:** Co-mutation of *SYNE1* and cancer-related genes (WT: wild-type, Mut: mutated).

Gene	*SYNE1* WT, N = 34 (%)	*SYNE1 Mut*, N = 16 (%)	*p*-Value ^1^	q-Value ^2^
*KMT2D*	8 (24%)	10 (62%)	0.007	0.1
*USH2A*	6 (18%)	8 (50%)	0.04	0.3
*BRCA2*	6 (18%)	7 (44%)	0.082	0.4
*FBXW7*	1 (2.9%)	2 (12%)	0.2	0.6
*PIK3CA*	5 (15%)	5 (31%)	0.3	0.6
*MSH6*	0 (0%)	1 (6.2%)	0.3	0.6
*CTNNB1*	2 (5.9%)	3 (19%)	0.3	0.8
*KRAS*	1 (2.9%)	1 (6.2%)	0.5	0.7
*MECOM*	3 (8.8%)	0 (0%)	0.5	0.7
*KMT2C*	10 (29%)	6 (38%)	0.6	0.7
*MSH2*	2 (5.9%)	2 (12%)	0.6	0.7
*BRCA1*	7 (21%)	4 (25%)	0.7	0.8
*PTEN*	6 (18%)	4 (25%)	0.7	0.8
*TP53*	17 (50%)	8 (50%)	>0.9	>0.9

^1^ Pearson’s Chi-squared test; Fisher’s exact test, ^2^ False discovery rate correction for multiple testing.

**Table 5:** Top 10 upregulated and downregulated genes in *SYNE1* mutated patients.

	Genes	logFC	*p*-Value	Q-Value	Functions
Upregulated					
	*ZCCHC12*	5.03431	1.19 × 10^−13^	4.46 × 10^−9^	Transcription regulation
	*DKK4*	8.38282	1.89 × 10^−12^	3.55 × 10^−8^	Cell-signaling
	*TPH1*	3.60935	7.84 × 10^−12^	9.79 × 10^−8^	Neurotransmitter biosynthesis
	*CCL21*	4.37155	4.33 × 10^−11^	4.06 × 10^−7^	Cytokine signaling
	*SLC14A1*	3.95827	1.12 × 10^−10^	8.37 × 10^−7^	Membrane transporter
	*TRH*	4.89343	2.51 × 10^−10^	1.57 × 10^−6^	Hormone signaling
	*MS4A1*	4.28194	1.84 × 10^−9^	9.85 × 10^−6^	Lymphocyte differentiation
	*C1QTNF9B*	3.37106	5.89 × 10^−9^	2.76 × 10^−5^	Cell-signaling
	*NIBAN3*	2.75499	9.52 × 10^−9^	3.96 × 10^−5^	Apoptosis regulator
	*TDRD15*	3.54089	3.36 × 10^−8^	1.25 × 10^−4^	Nucleic acid binding
Downregulated					
	*ACTC1*	−6.50242	4.26 × 10^−6^	3.63 × 10^−3^	Cell motility
	*RPL7AP9*	−2.5812	6.90 × 10^−6^	5.38 × 10^−3^	Pseudogene
	*RFX4*	−4.67237	7.48 × 10^−6^	5.61 × 10^−3^	Transcription regulation
	*KRT6C*	−7.43576	1.91 × 10^−5^	1.09 × 10^−2^	Structural integrity
	*ELAVL2*	−3.95196	2.33 × 10^−5^	1.23 × 10^−2^	Post-translational modifications
	*MTCO2P12*	−3.52321	2.42 × 10^−5^	1.24 × 10^−2^	Mitochondrial electron transport
	*KRT6B*	−6.06818	2.85 × 10^−5^	1.37 × 10^−2^	Cytoskeleton signaling
	*C4BPA*	−3.97177	3.93 × 10^−5^	1.71 × 10^−2^	Complement activation
	*AMY1C*	−3.70389	6.83 × 10^−5^	2.33 × 10^−2^	Amylase enzyme
	*PLAAT1*	−1.81246	6.89 × 10^−5^	2.33 × 10^−2^	Acetyltransferase activity

## Data Availability

Data from The Cancer Genome Atlas was used for this research study. A total of 523 ovarian cancer patients in the Ovarian Serous Cystadenocarcinoma cohort (TCGA, PanCancer Atlas) were used. The dataset can be accessed here: https://www.cbioportal.org/study/summary?id=ov_tcga_pan_can_atlas_2018, accessed on 12 April 2023.

## Supplementary Materials

The following supporting information can be downloaded at: https://www.mdpi.com/article/10.3390/ijms241814212/s1.
