# Supplementary material for: SYNE1 Mutation Is Associated with Increased Tumor Mutation Burden and Immune Cell Infiltration in Ovarian Cancer
Source: Int J Mol Sci. 2023 Sep 18;24(18):14212. doi: 10.3390/ijms241814212 (PMC10531966; doi:10.3390/ijms241814212)
Supplement: Supplementary file 1 [file ijms-24-14212-s001.zip › ijms-2550730-supplementary.pdf]

Supplementary Table S1. Details of *SYNE1* mutations among *SYNE1* mutated patients at The Markey Cancer Center. All mutations represent single nucleotide polymorphisms (SNP).

1	E3612*	Nonsense	152354751	152354751	C	A
	G6681C	Missense	152239559	152239559	C	A
2	S7253L	Missense	152220945	152220945	G	A
	R5623G	Missense	152310717	152310717	G	C
3	A2475E	Missense	152396907	152396907	G	T
4	L8595F	Missense	152135109	152135109	G	A
5	E5309G	Missense	152321878	152321878	T	C
6	R7157L	Missense	152224546	152224546	C	A
	I5417T	Missense	152319002	152319002	A	G
	D3894A	Missense	152350670	152350670	T	G
7	E2572Q	Missense	152391567	152391567	C	G
8	M706L	Missense	152462872	152462872	T	A
9	E8397G	Missense	152141259	152141259	T	C
10	C4048G	Missense	152344164	152344164	A	C
11	V7481A	Missense	152213664	152213664	A	G
	R5617*	Nonsense	152310735	152310735	G	A
12	Q565R	Missense	152466017	152466017	T	C
13	Q6528L	Missense	152244646	152244646	T	A
	T2659N	Missense	152391305	152391305	G	T
	K1076E	Missense	152450794	152450794	T	C
	R595K	Missense	152465406	152465406	C	T
14	Q6528L	Missense	152244646	152244646	T	A
	L1548Q	Missense	152430528	152430528	A	T
	K1076E	Missense	152450794	152450794	T	C

15	A3012E	Missense	152376887	152376887	G	T
16	H8716Q	Missense	152130725	152130725	A	C
	M1596L	Missense	152430114	152430114	T	G
	P1407L	Missense	152436031	152436031	G	A
17	Q3900*	Nonsense	152350653	152350653	G	A
	L3899I	Missense	152350656	152350656	G	T
18	*3324*	Intron	152367196	152367196	A	T
	3324	Intron	152367198	152367198	T	C
	S2519N	Missense	152396775	152396775	C	T
